# *Molecular Oncology* and the EACR: new partners in communicating and supporting cancer research

**DOI:** 10.1002/1878-0261.13445

**Published:** 2023-06-10

**Authors:** Kevin M. Ryan, Jane Smith, René Bernards

**Affiliations:** ^1^ Molecular Oncology Editorial Office FEBS Press Cambridge UK; ^2^ Cancer Research UK Beatson Institute Glasgow UK; ^3^ School of Cancer Sciences University of Glasgow UK; ^4^ European Association for Cancer Research Sir Colin Campbell Building Nottingham UK; ^5^ Division of Molecular Carcinogenesis, Oncode Institute The Netherlands Cancer Institute Amsterdam The Netherlands

## Abstract

The formation of organisations and societies within all areas of scientific research facilitates the bringing together of researchers in a given field and serves to aid communication, collaboration, progress of science and career development. Even greater gain can be attained when individual organisations form partnerships to complement each other's activities and to increase the scope of their endeavours. Within this editorial, we highlight the key points of a new partnership formed between two non‐profit bodies within cancer research, the European Association for Cancer Research (EACR) and *Molecular Oncology*, a journal wholly owned by the Federation of European Biochemical Societies (FEBS).

## Graphical abstract

The formation of organisations and societies facilitates bringing researchers together and aids communication, collaboration, scientific progress and career development. In this editorial, we highlight key points of a newly formed partnership between two charitable organisations within cancer research, the European Association for Cancer Research (EACR) and *Molecular Oncology*, a journal wholly owned by the Federation of European Biochemical Societies (FEBS).

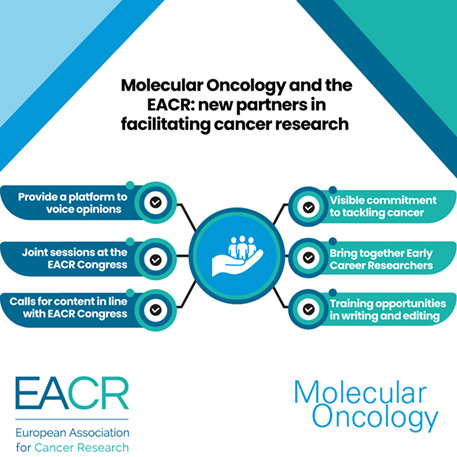

## 1 Background

The European Association for Cancer Research (EACR) was founded in 1968 to enable a greater collaboration between cancer researchers across geographical Europe (https://www.eacr.org/). The European Association for Cancer Research's mission focusses on ‘The advancement of cancer research for the public benefit: from basic research to prevention, treatment and care’. The organisation has gone from strength to strength since its inception and now has over 12 000 members worldwide. It continues to promote in‐person and virtual collaboration and networking between cancer researchers, as well as offering a variety of funding opportunities to support its members. For example, the EACR supports travel fellowships and travel awards that assist early career researchers in gaining greater experience by visits to other laboratories or by attending scientific events. The EACR itself also organises a number of scientific events throughout the year, with the annual EACR Congress being the association's landmark event. The 2023 EACR Congress is the 29th annual congress and will take place in Torino, Italy, between the 12th and 15th of June.

Similar to the EACR, the Federation of European Biochemical Societies (FEBS), founded in 1964, is a non‐profit society focussed on biochemistry and molecular biology (https://www.febs.org/). The Federation of European Biochemical Societies is made up of 39 Constituent Societies across Europe and nearby states, which culminates in a membership of more than 35 000 researchers. The Federation of European Biochemical Societies is supported by FEBS Press, which publishes four journals: *FEBS Journal, FEBS Letters, FEBS Open Bio and Molecular Oncology. Molecular Oncology* is a multidisciplinary cancer journal publishing basic, translational and clinical cancer research as well as policy articles reporting developments in the cancer research community. All profits from the four journals are reinvested back into science to support fellowships, travel grants, FEBS Advanced Courses, the annual FEBS Congress (https://2023.febscongress.org/) and other FEBS Initiatives.

## 2 Recent developments

Initial interactions between *Molecular Oncology* and the EACR began during 2022 when members of the editorial office attended and promoted various conferences organised by the EACR. This connection was also extended during the 2022 EACR Congress, when members of the *Molecular Oncology* editorial office actively participated and supported the congress via various initiatives such as participation in the ‘Meet the Editor’ Session. The highlight of this collaboration at the 2022 EACR Congress was a session on science policy that was jointly organised by *Molecular Oncology*, the EACR and the European Academy of Cancer Sciences (EACS; https://www.europeancanceracademy.eu/). Due to this session's great success and since the EACR and FEBS have similar goals in supporting science, further discussions ensued to see whether there could be additional common goals between the EACR and *Molecular Oncology* that they could work together towards. This culminated in a memorandum of understanding between *Molecular Oncology*, FEBS and the EACR covering various points that led to the recognition of *Molecular Oncology* as an ‘Affiliate Journal’ of the EACR.

Following the success of the joint *Molecular Oncology*/EACR/EACS session at the 2022 EACR Congress, it was agreed to hold a second joint session at the 2023 Congress on the topic of ‘Challenges of Data Management and Sharing for Translational Cancer Research’ [1]. Members of the *Molecular Oncology* editorial office will also be in attendance at the Congress, and Kevin Ryan, Editor‐in‐Chief of *Molecular Oncology*, will take part in a ‘Meet the Editors’ panel discussion together with editors and publishers from other cancer journals . *Molecular Oncology* will also publish the abstract proceedings of the EACR Congress as a supplement in a special issue.

In further connections between the EACR and *Molecular Oncology*, the journal has recently started to publish a new article type entitled ‘Viewpoints from the EACR’, in which researchers connected to the EACR are invited to write commentaries on recent developments or contentious topics in cancer research and their transformative opinions. Two of these have already been published [2, 3], and the aim is for this to be a recurring feature in future issues.

In recognition of the fact that both FEBS and the EACR have a central focus on supporting early career researchers, *Molecular Oncology* will also form an Editorial Academy inviting EACR early career members to gain experience in publishing and journal editing. Moreover, the *Molecular Oncology* editorial office will also organise a small number of publishing internships to further support this goal by hosting EACR members as interns at the journal to familiarise themselves with the other side of peer review. Further collaborations between the EACR Early Career Researchers' Council and the *Molecular Oncology* Early Career Advisory Board will also be encouraged and developed.

The aim of this partnership between the EACR and Molecular Oncology is to strengthen collaboration and facilitate progress in cancer research within Europe and beyond. Both parties have agreed to promote each other's individual, as well as joint initiatives, and all involved hope that this collaboration grows and brings great positivity in the years to come.

## Conflict of interest

The authors declare no conflict of interest.
